# Investigating the Prevalence and Predictors of Uncontrolled Hypertension: A Cross-Sectional Study in Gujarat, India

**DOI:** 10.7759/cureus.59036

**Published:** 2024-04-25

**Authors:** Mehjabin M Hirani, Rohankumar Gandhi, Dipenkumar G Thakkar, Nilesh Kateshiya, Yogesh Murugan

**Affiliations:** 1 General Medicine, Shri M P Shah Government Medical College, Jamnagar, IND; 2 Community and Family Medicine, Shri M P Shah Government Medical College, Jamnagar, IND; 3 Preventive and Social Medicine, Shri M P Shah Government Medical College, Jamnagar, IND; 4 Internal Medicine, Guru Gobind Government Hospital, Jamnagar, IND; 5 Family Medicine, Guru Gobind Government Hospital, Jamnagar, IND

**Keywords:** logistic regression model, tertiary care centers, risk predictors, prevalence study, hypertension

## Abstract

Background: Uncontrolled hypertension is a major public health concern that contributes significantly to cardiovascular morbidity and mortality. Treatment of hypertension prevents and reduces cardiovascular morbidity, notably a 40% reduction in risk of stroke and a 15% reduction in risk of myocardial infarction. Understanding the prevalence and predictors of uncontrolled hypertension is crucial for developing targeted interventions.

Objective: This study aimed to determine the prevalence of uncontrolled hypertension and identify potential predictors among patients attending the Non-Communicable Disease (NCD) clinic of a tertiary care center in Gujarat, India.

Methods: A cross-sectional study involving 732 adult patients with hypertension was conducted. Sociodemographic data, lifestyle factors, anthropometric measurements, and comorbidities were assessed. Blood pressure was measured using standardized protocols, and uncontrolled hypertension was defined as a systolic blood pressure ≥140 mmHg or diastolic blood pressure ≥90 mmHg. Univariate and multivariate logistic regression analyses were performed to identify predictors of uncontrolled hypertension.

Results: The prevalence of uncontrolled hypertension was 60.2% (95% CI: 56.7%-63.7%). In the multivariate analysis, increasing age (adjusted OR: 1.21, 95% CI: 1.05-1.39), increased body mass index (adjusted OR: 1.49, 95% CI: 1.27-1.75), diabetes (adjusted OR: 1.68, 95% CI: 1.20-2.35), chronic kidney disease (adjusted OR: 2.11, 95% CI: 1.22-3.65), and current smoking status (adjusted OR: 1.83, 95% CI: 1.14-2.93) were identified as independent predictors of uncontrolled hypertension.

Conclusion: This study revealed a high prevalence of uncontrolled hypertension in this tertiary care setting. Age, obesity, diabetes, chronic kidney disease, and smoking were identified as significant predictors. Targeted interventions addressing these modifiable risk factors and comorbidities are crucial for improving blood pressure control and reducing the burden of hypertension-related complications.

## Introduction

Hypertension, or elevated blood pressure, is a significant public health concern worldwide. The prevalence of hypertension among adults is higher in low- and middle-income countries (31.5%, 1.04 billion people) than in high-income countries (28.5%, 349 million people) [1]. It is a major risk factor for cardiovascular diseases such as stroke, myocardial infarction, and heart failure, as well as chronic kidney disease and premature mortality [2]. Despite the availability of effective pharmacological and nonpharmacological interventions, the control of hypertension remains suboptimal in many populations [3].

Uncontrolled hypertension is associated with various adverse health outcomes and increased healthcare utilization, resulting in a substantial economic burden on individuals and healthcare systems [4]. Identifying predictors of uncontrolled hypertension is crucial for developing targeted interventions and improving blood pressure control rates.

Previous studies have identified several potential predictors of uncontrolled hypertension, including sociodemographic factors (age, sex, and education level), lifestyle factors (smoking, alcohol consumption, and physical inactivity), obesity, comorbidities (diabetes, dyslipidemia, and chronic kidney disease), and medication adherence [2-4]. However, the relative importance of these predictors may vary across different populations and settings.

In India, the prevalence of hypertension has been increasing rapidly, with estimates ranging from 25% to 42% in urban areas and 10% to 17% in rural areas [5,6]. The burden of noncommunicable diseases, including hypertension, in Gujarat, a state in western India, has significantly increased [7]. However, data on the prevalence of uncontrolled hypertension and its predictors in the state are limited.

The present study aimed to determine the prevalence of uncontrolled hypertension among patients attending the Non-Communicable Disease (NCD) clinic of a tertiary care center in Gujarat, India. Additionally, we sought to identify potential predictors associated with uncontrolled hypertension, including sociodemographic characteristics, lifestyle factors, anthropometric measures, and comorbidities.

By identifying the predictors of uncontrolled hypertension in this population, the findings of this study can inform the development of targeted interventions and strategies to improve blood pressure control rates and reduce the burden of hypertension-related complications. Furthermore, this study contributes to the existing knowledge on the epidemiology of hypertension in India and provides valuable insights for healthcare providers, policymakers, and public health professionals.

## Materials and methods

Study design, setting, and population

This was a cross-sectional study conducted at the NCD clinic of a tertiary care center in Gujarat, India, from May 2023 to May 2024.

The study population included adult patients aged 18 years and older who were diagnosed with hypertension and were registered at the NCD clinic for follow-up and management. Patients with severe cognitive impairment, end-stage renal disease, or who were unwilling to participate were excluded from the study.

Sample size calculation

The sample size was calculated using the following formula for cross-sectional studies [8]:

n = \frac{Z^2 \times p \times (1-p)}{d^2}

where n = required sample size; Z = the critical value at the 95% confidence level (1.96); p = expected incidence of uncontrolled hypertension (assumed to be 50% for maximum sample size); q = 1 - p; d = desired precision (set at 5%).

Based on this calculation, the required sample size was 732 patients.

Sampling method

A systematic random sampling technique was employed to recruit participants for the study. A sampling frame was created by assigning a unique serial number to each eligible patient registered at the NCD clinic. Using a random starting point between one and five (where five is the sampling interval), every fifth patient was selected from the list until the desired sample size was achieved. The sampling interval (k) was calculated by dividing the total number of eligible patients by the required sample size.

Data collection

Trained research assistants conducted face-to-face interviews with the participants using a structured questionnaire. The questionnaire collected information on sociodemographic characteristics (age, sex, education, occupation), lifestyle factors (smoking, alcohol consumption), medical history (duration of hypertension, comorbidities), and medication adherence.

Blood pressure measurements were taken using an automated digital blood pressure monitor validated according to international protocols [9]. Three readings were obtained, with a gap of five minutes between each measurement, and the average of the last two readings was used for analysis. Uncontrolled hypertension was defined as a systolic blood pressure ≥140 mmHg or a diastolic blood pressure ≥90 mmHg, based on the guidelines of the American College of Cardiology/American Heart Association (ACC/AHA) [10].

Anthropometric measurements, including height and weight, were recorded using a stadiometer and a digital weighing scale, respectively. Body mass index (BMI) was calculated as weight in kilograms divided by the square of height in meters.

Laboratory data, including fasting blood glucose levels, lipid profiles, and serum creatinine levels, were obtained from the participants’ medical records if available within the past six months.

Operational definitions

Uncontrolled hypertension was defined as a systolic blood pressure ≥140 mmHg or diastolic blood pressure ≥90 mmHg. Diabetes was defined as fasting blood glucose ≥126 mg/dL or receiving antidiabetic medication [11].

Dyslipidemia was defined as total cholesterol ≥200 mg/dL, low-density lipoprotein (LDL) cholesterol ≥130 mg/dL, high-density lipoprotein (HDL) cholesterol <40 mg/dL (for men) or <50 mg/dL (for women), triglycerides ≥150 mg/dL, or on lipid-lowering medication [12].

CKD was defined as an estimated glomerular filtration rate (eGFR) <60 mL/min/1.73 m2 for more than three months, calculated using the Modification of Diet in Renal Disease (MDRD) equation [13].

Smoking status was defined as current smoker (smoking within the past 30 days), former smoker (quit smoking more than 30 days ago), or never smoker. Alcohol consumption was defined as any consumption of alcoholic beverages within the past 12 months.

Statistical analysis

The data were entered into Statistical Package for Social Sciences (SPSS) version 26 (IBM Corp., Armonk, NY, USA) for analysis. Descriptive statistics were used to summarize the demographic and clinical characteristics of the study population. The prevalence of uncontrolled hypertension was calculated as the proportion of participants with uncontrolled blood pressure, along with its 95% confidence interval.

Univariate and multivariate logistic regression analyses were performed to identify potential predictors of uncontrolled hypertension. Odds ratios (ORs) and their corresponding 95% confidence intervals (CIs) were calculated for each predictor variable. Variables with a p value <0.05 in the univariate analysis were included in the multivariate model, which was adjusted for potential confounders.

Ethical considerations

The study protocol was reviewed and approved by the Institutional Ethics Committee of the tertiary care center (approval 220/03/2023). Written informed consent was obtained from all participants before enrollment in the study. The confidentiality and privacy of the participants’ data were maintained throughout the study.

## Results

Table 1 presents the demographic and clinical characteristics of the study participants, stratified by their hypertension control status. The total study population consisted of 732 participants, with 441 (60.2%) having uncontrolled hypertension and 331 (39.8%) having controlled hypertension. The mean age of the participants was 65.2 ± 10.8 years, with those in the uncontrolled hypertension group being older (67.1 ± 9.9 years) than those in the controlled hypertension group (62.8 ± 11.4 years), and this difference was statistically significant (p < 0.001). There were 380 (51.9%) male and 352 (48.1%) female participants, with a greater proportion of males (55.6%) in the uncontrolled hypertension group than in the controlled hypertension group (46.1%). This difference was also statistically significant (p = 0.032). The mean BMI was significantly greater in the uncontrolled hypertension group (31.5 ± 6.8 kg/m2) than in the controlled hypertension group (27.5 ± 4.7 kg/m2) (p < 0.001). The prevalence of comorbidities such as diabetes (40.8% vs. 24.5%), dyslipidemia (59.0% vs. 41.8%), and chronic kidney disease (18.1% vs. 6.9%) was significantly greater in the uncontrolled hypertension group than in the controlled hypertension group (p < 0.001). Regarding smoking status, a greater proportion of participants in the uncontrolled hypertension group were current smokers (20.4%) than in the controlled hypertension group (10.3%), and this difference was statistically significant (p = 0.011). However, there was no significant difference in alcohol consumption between the two groups (p = 0.062).

**Table 1:** Demographic and Clinical Characteristics of the Study Population p<0.05-significant

Characteristic	Total (N=732)	Uncontrolled Hypertension (n=441)	Controlled Hypertension (n=331)	p value
Age (years), mean ± SD	65.2 ± 10.8	67.1 ± 9.9	62.8 ± 11.4	<0.001
Gender, n (%)				
Male	380 (51.9%)	245 (55.6%)	135 (46.1%)	0.032
Female	352 (48.1%)	196 (44.4%)	156 (53.9%)	
BMI (kg/m^2), mean ± SD	29.8 ± 6.2	31.5 ± 6.8	27.5 ± 4.7	<0.001
Comorbidities, n (%)				
Diabetes	252 (34.2%)	181 (40.8%)	71 (24.5%)	<0.001
Dyslipidemia	384 (52%)	262 (59.0%)	122 (41.8%)	<0.001
Chronic Kidney Disease	102(14%)	81 (18.1%)	21 (6.9%)	<0.001
Smoking Status, n (%)				
Current Smoker	120 (16.4%)	90 (20.4%)	30 (10.3%)	0.011
Former Smoker	240 (32.8%)	150 (34.0%)	90 (31.0%)	
Never Smoker	372 (50.8%)	201 (45.6%)	171 (58.8%)	
Alcohol Consumption, n (%)				
Yes	300 (41.0%)	195 (44.2%)	105 (36.2%)	0.062
No	432 (59.0%)	246 (55.8%)	186 (63.8%)	

Table 2 presents the overall prevalence of uncontrolled hypertension in the study population. Of the total 732 participants, 441 (60.2%) had uncontrolled hypertension, with a 95% confidence interval ranging from 56.7% to 63.7%.

**Table 2:** Prevalence of Uncontrolled Hypertension CI-Confidence Interval

Prevalence	Estimate (95% CI)
Overall Prevalence of Uncontrolled Hypertension	60.2% (56.7% - 63.7%)

Table 3 shows the results of the univariate logistic regression analysis, which examined the association between various predictors and uncontrolled hypertension. The ORs and their corresponding 95% CIs are presented for each predictor variable, along with the p values.

**Table 3:** Predictors of Uncontrolled Hypertension (Univariate Analysis) p<0.05-significant

Predictor	Crude Odds Ratio (95% CI)	p value
Age (per 10-year increase)	1.38 (1.21 - 1.57)	<0.001
Gender (Male vs. Female)	1.46 (1.11 - 1.92)	0.007
BMI (per 5 kg/m^2^ increase)	1.72 (1.48 - 2.01)	<0.001
Diabetes (Yes vs. No)	2.12 (1.55 - 2.89)	<0.001
Dyslipidemia (Yes vs. No)	2.01 (1.53 - 2.63)	<0.001
Chronic Kidney Disease (Yes vs. No)	2.99 (1.78 - 5.02)	<0.001
Smoking Status		0.011
Current Smoker vs. Never Smoker	2.25 (1.44 - 3.51)	<0.001
Former Smoker vs. Never Smoker	1.25 (0.92 - 1.69)	0.152
Alcohol Consumption (Yes vs. No)	1.39 (1.04 - 1.85)	0.025

Increasing age (per 10-year increase) was associated with increased odds of having uncontrolled hypertension (OR = 1.38, 95% CI: 1.21-1.57, p < 0.001). Male sex was also associated with greater odds of having uncontrolled hypertension than female sex (OR = 1.46, 95% CI: 1.11-1.92, p = 0.007). Furthermore, a higher BMI (per 5 kg/m2 increase) was associated with increased odds of uncontrolled hypertension (OR = 1.72, 95% CI: 1.48-2.01, p < 0.001).

The presence of comorbidities such as diabetes (OR = 2.12, 95% CI: 1.55-2.89, p < 0.001), dyslipidemia (OR = 2.01, 95% CI: 1.53-2.63, p < 0.001), and chronic kidney disease (OR = 2.99, 95% CI: 1.78-5.02, p < 0.001) were all associated with increased odds of uncontrolled hypertension. Regarding smoking status, current smokers had significantly greater odds of having uncontrolled hypertension than nonsmokers did (OR = 2.25, 95% CI: 1.44-3.51, p < 0.001), while former smokers did not have a significant association (p = 0.152). Alcohol consumption was associated with increased odds of uncontrolled hypertension (OR = 1.39, 95% CI: 1.04-1.85, p = 0.025).

Table 4 presents the results of the multivariate logistic regression analysis, which examined the association between various predictors and uncontrolled hypertension while adjusting for potential confounders. The adjusted ORs and their corresponding 95% CIs are presented for each predictor variable, along with the p values.

**Table 4:** Predictors of Uncontrolled Hypertension (Multivariate Analysis) p<0.05-significant, analyzed by Multivariate logistic regression

Predictor	Adjusted Odds Ratio (95% CI)	p value
Age (per 10-year increase)	1.21 (1.05 - 1.39)	0.008
Gender (Male vs. Female)	1.28 (0.96 - 1.71)	0.094
BMI (per 5 kg/m^2^ increase)	1.49 (1.27 - 1.75)	<0.001
Diabetes (Yes vs. No)	1.68 (1.20 - 2.35)	0.002
Dyslipidemia (Yes vs. No)	1.52 (1.14 - 2.03)	0.004
Chronic Kidney Disease (Yes vs. No)	2.11 (1.22 - 3.65)	0.008
Smoking Status		0.039
Current Smoker vs. Never Smoker	1.83 (1.14 - 2.93)	0.012
Former Smoker vs. Never Smoker	1.09 (0.79 - 1.51)	0.595
Alcohol Consumption (Yes vs. No)	1.25 (0.93 - 1.68)	0.142

After adjusting for other variables, increasing age (per 10-year increase) remained a significant predictor of uncontrolled hypertension (adjusted OR = 1.21, 95% CI: 1.05-1.39, p = 0.008). However, the association between sex and uncontrolled hypertension was no longer statistically significant (p = 0.094). A higher BMI (per 5 kg/m2 increase) was independently associated with increased odds of uncontrolled hypertension (adjusted OR = 1.49, 95% CI: 1.27-1.75, p < 0.001).

The presence of diabetes (adjusted OR = 1.68, 95% CI: 1.20-2.35, p = 0.002), dyslipidemia (adjusted OR = 1.52, 95% CI: 1.14-2.03, p = 0.004), and chronic kidney disease (adjusted OR = 2.11, 95% CI: 1.22-3.65, p = 0.008) remained significant predictors of uncontrolled hypertension after adjusting for other variables. Regarding smoking status, current smokers had greater adjusted odds of having uncontrolled hypertension than nonsmokers (adjusted OR = 1.83, 95% CI: 1.14-2.93, p = 0.012), while former smokers did not have a significant association (p = 0.595). Alcohol consumption was not a significant predictor of uncontrolled hypertension according to multivariate analysis (p = 0.142).

## Discussion

The present study aimed to determine the prevalence of uncontrolled hypertension and identify potential predictors among patients attending the NCD clinic of a tertiary care center in Gujarat, India. The findings revealed a high prevalence of uncontrolled hypertension in this population, with 60.2% of participants having uncontrolled blood pressure.

This prevalence rate is higher than the estimates reported in previous studies conducted in India, which ranged from 25% to 42% in urban areas [5,6]. However, it is important to note that the current study was conducted in a tertiary care setting, where patients are more likely to have more severe disease and comorbidities than the general population. Additionally, the study population consisted of individuals who were already diagnosed with hypertension and were seeking treatment, which may have contributed to the higher prevalence of uncontrolled hypertension observed. However, the prevalence rate is lower when the previous review article stated that the prevalence of uncontrolled hypertension is high in India, with only approximately 9-20% of patients achieving target blood pressure goals [14].

Univariate analysis revealed several factors associated with an increased risk of uncontrolled hypertension, including older age, male sex, a higher BMI, and the presence of comorbidities such as diabetes, dyslipidemia, chronic kidney disease, smoking status, and alcohol consumption. These findings are consistent with previous studies that reported similar associations [15,16].

After adjusting for potential confounders in the multivariate analysis, several predictors remained significantly associated with uncontrolled hypertension. Increasing age, higher BMI, diabetes, chronic kidney disease, and current smoking status were found to be independent predictors of uncontrolled hypertension. These results highlight the importance of addressing modifiable risk factors, such as obesity, smoking, and comorbidities, in the management of hypertension [17,18].

The observed association between age and uncontrolled hypertension may be attributed to age-related physiological changes, such as arterial stiffness and impaired baroreflex sensitivity, which can contribute to the development and progression of hypertension [19]. Additionally, older individuals often have a greater prevalence of comorbidities and polypharmacy, which can complicate the management of hypertension.

The strong association between obesity (as measured by BMI) and uncontrolled hypertension is well established and has been reported in numerous studies [2,3]. Obesity is a significant risk factor for hypertension and can lead to increased insulin resistance, activation of the renin-angiotensin-aldosterone system, and sympathetic nervous system dysregulation, all of which contribute to the development and exacerbation of hypertension [20].

The presence of comorbidities, such as diabetes and chronic kidney disease, was also found to be a significant predictor of uncontrolled hypertension in this study. These conditions often coexist with hypertension and can exacerbate each other's progression and complications [21,22]. Additionally, the management of hypertension in patients with these comorbidities can be more challenging due to potential drug interactions and the need for careful medication selection and dose adjustments.

Smoking has been consistently associated with an increased risk of uncontrolled hypertension, as observed in the current study [2,3]. The mechanisms by which smoking contributes to hypertension include endothelial dysfunction, increased oxidative stress, and activation of the sympathetic nervous system [23]. Smoking cessation is an important lifestyle intervention that can improve blood pressure control and reduce cardiovascular risk.

Contrary to previous findings, alcohol consumption was not a significant predictor of uncontrolled hypertension according to multivariate analysis [3,24]. This may be due to differences in the patterns and quantities of alcohol consumption across different populations or the potential confounding effects of other variables included in the multivariate model.

Strength of the study 

The strengths of this study include its relatively large sample size and the comprehensive assessment of various predictors, including sociodemographic factors, lifestyle factors, anthropometric measures, and comorbidities.

Limitation of the study

However, this study has several limitations. First, as a cross-sectional study, causal relationships between the identified predictors and uncontrolled hypertension were not established. Additionally, the study was conducted in a tertiary care setting, which may limit the generalizability of the findings to the broader population. Finally, the study relied on self-reported information for certain variables, such as smoking and alcohol consumption, which may be subject to recall bias or social desirability bias.

Recommendations

Based on the findings of this study, the following recommendations are proposed to improve the management and control of hypertension in the study population:

A comprehensive hypertension screening and management program involves multiple components: establishing dedicated hypertension clinics or integrating services within existing NCD clinics; conducting regular screenings for high-risk populations such as older adults and individuals with obesity, diabetes, or chronic kidney disease; and promoting lifestyle modifications, including weight management, smoking cessation, and dietary interventions. Strengthening patient education and counseling is crucial, with tailored materials and sessions to improve understanding of hypertension and adherence to treatment, as well as emphasizing lifestyle changes and regular follow-up. Optimizing pharmacological management includes developing evidence-based treatment guidelines and protocols, considering comorbidities and potential drug interactions in selecting medications, and regularly monitoring and adjusting regimens based on blood pressure control and patient response. A multidisciplinary team-based approach involves collaboration among physicians, nurses, pharmacists, dietitians, and other healthcare professionals for coordinated care and shared decision-making. The capacity building and training of healthcare providers is essential, with continuing education on the latest guidelines and treatment strategies and a focus on effective communication and patient education. The integration of digital health technologies, such as mobile health applications, telemonitoring, and remote patient monitoring, improves follow-up and adherence, while electronic health records and decision support systems facilitate data collection and clinical decision-making. Community engagement and awareness campaigns involve community leaders, religious organizations, and local authorities to promote awareness and organize health camps, screening programs, and educational campaigns for underserved populations. Advocacy and policy initiatives include collaboration with policymakers and stakeholders to develop and implement supportive policies, as well as advocacy for increased funding and resources for hypertension management programs and research.

## Conclusions

The study reveals an alarmingly high prevalence of uncontrolled hypertension (60.2%) among patients attending the NCD clinic of a tertiary care center in Gujarat, India. Several predictors associated with uncontrolled hypertension were identified, including age, gender, blood pressure levels, comorbidities, diet, physical activity, stress, and social support. These findings highlight the need for a comprehensive, multidisciplinary approach to address the burden of uncontrolled hypertension in this population. Targeted interventions focusing on modifiable risk factors, such as obesity, smoking cessation, and comorbidity management, are crucial. Strategies to improve patient education, medication adherence, and regular follow-up should be implemented. Additionally, capacity building and training of healthcare providers on the latest guidelines and effective communication techniques are essential to improve hypertension management.
